# Chairwork in schema therapy for patients with borderline personality disorder—A qualitative study of patients' perceptions

**DOI:** 10.3389/fpsyt.2023.1180839

**Published:** 2023-06-02

**Authors:** Anna Katharina Josek, Anja Schaich, Diana Braakmann, Nele Assmann, Kamila Jauch-Chara, Arnoud Arntz, Ulrich Schweiger, Eva Fassbinder

**Affiliations:** ^1^Department of Psychiatry and Psychotherapy, University of Lübeck, Lübeck, Germany; ^2^Department of Psychiatry and Psychotherapy, Christian-Albrechts Universität Kiel, Kiel, Germany; ^3^Department of Clinical Psychology, University of Amsterdam, Amsterdam, Netherlands; ^4^Department of Psychiatry, Psychosomatics and Psychotherapy, University of Lübeck, Lübeck, Germany

**Keywords:** borderline personality disorder, chairwork, qualitative research, schema therapy, perspective, experiential techniques, psychotherapy

## Abstract

**Objective:**

Chairwork is one of the core experiential techniques of Schema Therapy (ST) which is used in the treatment of patients with borderline personality disorder (BPD). However, little is known about how people with BPD experience chairwork. The aim of this study was to explore the experiences of patients with BPD with chairwork in ST.

**Method:**

Qualitative data were collected through semi-structured interviews with 29 participants with a primary diagnosis of BPD who experienced chairwork as part of their ST treatment. The interview data were analyzed using qualitative content analysis.

**Findings:**

Many participants reported initial skepticism, and difficulties with engaging in chairwork. Specific therapist behaviors as well as some external (e.g., restricted facilities, noise) and internal factors (especially feeling ashamed or ridiculous) were named as hindering factors. Participants described several therapist behaviors facilitating chairwork such as providing safety, clear guidance through the process as well as flexible application of the technique according to their needs, and sufficient time for debriefing. Participants experienced emotional pain and exhaustion as short-term effects of the technique. All participants reported positive long-term effects including an improved understanding of their mode model as well as positive mode changes (e.g., less Punitive Parent and more Healthy Adult Mode), greater self-acceptance, improvements in coping with emotions and needs as well as improvements in interpersonal relationships.

**Conclusions:**

Chairwork is experienced as an emotionally demanding but valuable technique. Based on the participants' statements, the delivery of chairwork can be optimized which can help to improve treatment outcome.

## 1. Introduction

Schema Therapy (ST) has been found to be an effective treatment for patients with borderline personality disorder (BPD) (1–3) as well as for individuals with other personality disorders (PD) (4). Qualitative studies also show that patients with BPD and other PDs value ST and its techniques as well as the benefits gained through treatment (5, 6). However, these studies were broad in scope and asked about patients' experiences with ST in general. Yet, ST is a complex treatment with multiple treatment techniques and various potential mechanisms that might lead to therapeutic gains. Detailed information about patients' experiences with specific ST techniques and their relationship to the change of mode processes is limited. Nevertheless, previous qualitative research suggest that experiential techniques are named by patients as among the most valuable techniques (6).

Therefore, we decided to use more specific questioning and conducted a research series focusing on experiential techniques which are core features of ST. Imaginary Rescripting (IR) was first investigated in the series (7) and we found various long-term effects of IR reported by participants, including a better understanding of schema modes and an improvement regarding emotion regulation, identifying, and dealing with schema modes and interpersonal relationships. We could identify hindering and facilitating factors for the implementation of IR based on which we were able to derive clinical implications for therapists for optimizing IR. The following article focusses on participants' experiences with chairwork, the other important experiential technique of ST, which has not yet been investigated in detail.

In ST, chairwork plays an important role in working with so-called “schema modes.” Most often these different sides of the patient are placed on different chairs and dialogues between them are initiated (8). Kellogg and Garcia Torres (9) describe The Four Dialogues as a framework to be used to categorize chairwork in a 2 x 2 matrix including internal/external and one chair/many chairs. Especially an internal orientation is widely used in ST, for example “interviewing” one schema mode (one chair) and mode dialogues (multiple chairs).

The following “schema modes” are characteristic of BPD: the Abandoned/Abused Child Mode (associated with strong emotions, such as sadness, loneliness, and fears of abandonment); the Angry, Impulsive Child Mode (reflected in angry outbursts, hostility or impulsive behaviors); the Punitive Parent Mode (characterized by self-hatred, shame, self-devaluation, and self-punishment); the Detached Protector Mode (associated with attempt to detach from emotional pain by maintaining distance from other people and avoiding or distracting from emotions with e.g., self-harm, dissociation, substance abuse, binge eating, or social withdrawal); and the Healthy Adult Mode (related to healthy functioning and relationships), which is often only barely present at the beginning of therapy. In treatment, specific tasks are pursued for every mode: care for the Vulnerable Child Modes to meet frustrated needs, help the Angry Child Modes deal with anger, combat the Punitive Parent and reassure the Detached Protector Mode, so that the patients can reduce their avoidance strategies and learn healthier strategies for managing emotions and relationships. Ultimately, the most important goal is to strengthen the Healthy Adult Mode.

Chairwork is assumed to be one of the core techniques to promote these changes on an experiential level. It can be used to better understand patients' problems in the light of the mode model and should help patients to experience emotions and needs in a safe way. Chairwork also aims to enable the patient to create new emotional experiences and to achieve changes in dysfunctional schemas and modes. To accomplish this, the therapist or the Healthy Adult Mode interacts with the other modes to adapt their statements and their actions to the above-named mode-specific goals of ST (e.g., comforting the Vulnerable Child, fighting the Punitive Parent Mode). This approach allows the patient to experience in a highly emotional way that their needs and feelings are important and that the self-devaluation typical for the Punitive Parent Mode can be reduced (8). Notably, all qualitative studies in ST have shown that patients experience experiential techniques as emotionally painful and demanding, yet important for their therapeutic gains (5–7). Detailed qualitative insights into patients' experiences with chairwork may help to optimize the application and to enhance patients' acceptance of this central ST technique as well as to better understand its effects.

Therefore, this study used content analysis of qualitative interviews with BPD patients diagnosed with BPD receiving chairwork in the context of ST to explore the following research questions:

1. What are BPD patients' experiences with chairwork in ST?
2. Which factors do BPD patients perceive as helpful or hindering regarding chairwork?
3. Which long- and short-term effects do the patients experience after receiving chairwork in ST?

## 2. Materials and methods

For the description of our research design, approach to data acquisition and analysis as well as the presentation of our findings, we follow the reporting standards for journal articles for qualitative research (10).

### 2.1. Recruitment and participants

Data were collected by interviewing 29 patients with BPD. Patients were recruited from the ST condition of the PRO^*^BPD-trial, a randomized trial comparing the effectiveness of ST and Dialectical Behavior Therapy (DBT) for outpatients with BPD (11). This study was conducted at the outpatient clinic of the Department of Psychiatry and Psychotherapy, University of Lübeck, Germany. Main inclusion criteria for the PRO^*^BPD trial were a primary diagnosis of BPD and age between 18 and 65 years. Exclusion criteria were lifetime psychotic disorder, an IQ under 85, and acute severe substance dependence needing clinical detoxification treatment. Additional information on recruitment and procedures of the PRO^*^BPD study can be found in the study protocol (12).

Patients participating in PRO^*^BPD were contacted to take part in the qualitative study if they had received at least 5 months of ST, were willing to participate in the qualitative study and gave their informed consent. At the time of the interview 26 participants had received at least 6 months of therapy, one had already completed therapy (early success) and two had dropped out.

Table 1 gives demographic and clinical characteristics of this study's sample.

**Table 1:** Demographic and clinical characteristics of the sample (*N* = 29).

	***n***	**%**
**Gender**		
Male	6	20.7
Female	23	79.3
**Highest education level**		
Elementary school (4 years)	1	3.4
Secondary school (9 years)	2	6.9
Secondary school (10 years)	13	44.8
Secondary school (12 years)	5	17.2
Secondary school (13 years)	4	13.8
Professional school	1	3.4
University degree	3	10.3
**Employment status**		
Student	5	17.2
Hommaker	1	3.4
Employed	6	20.7
Unemployed	1	3.4
Incapacitated for work	16	55.2
**Comorbid disorders (DSM-IV) Axis I**		
Affective disorders	19	65.5
Substance disorders	7	24.1
Anxiety disorders	25	86.2
Somatooform disorders	3	10.3
Eating disorders	15	51.7
**Axis II**		
Avoidant personality disorder	11	37.9
Obsessive compulsive personality disorder	13	44.8
Dependent personality disorder	4	13.8
Paranoid personality disorder	4	13.8
Schizotypal personality disorder	2	6.9
Histrionic personality disorder	2	6.9
Narcissistic personality disorder	3	10.3

### 2.2. Procedure and data collection

After receiving verbal and written explanation of the study all subjects provided written informed consent. The Ethics Committee of the University of Lübeck approved of the research protocol and amendments.

We used in-depth semi-structured interviews (“Qualitative Interview for techniques in schema therapy part 1 (Imagery Rescripting) and part 2 (Chairwork)“) developed by three of the authors (EF, AA, US) who have extensive experience in BPD research and treatment. The part of the interview relevant for the present study (part 2 Chairwork) can be found in Table 2. The interviews began with an open question regarding the participants' general experiences with chairwork. Follow up questions (compare italics in Table 2) helped the participants to elaborate on the question and to address specific issues. The interviews were conducted by four graduate students in psychology who were not involved in the treatment delivery and had no information about the study outcome. Two questions were added later (compare underlined items in Table 2) and assessed by telephone for the interviews already conducted.

**Table 2:** Qualitative interview on components of schematherapy—Chairwork.

Now, we are going to focus on Chairwork
What are your experiences with Chairwork? • *Positive and negative experiences* • *What did you find particularly easy or difficult?* • *Costs and benefits of Chairwork*
What did you find helpful or less helpful about the behavior of your therapist during the performance of the technique? *If not addressed during free speech:* • *Introduction of the technique* • *Performance of the technique* • *Debriefing* • *Time management* • *Emotional support by the therapist* Were there other factors that you found helpful or hindering regarding the performance of the technique? For example, external or internal factors such as thoughts or emotions? • *Regarding your modes?*
Which effects did you notice after the performance of the technique? • *If not addressed during free speech:* • *Short-term, long-term?* • *Regarding your interpersonal relationships?* • *Regarding the way you look at yourself?* • *Emotional? Cognitive? Physical? Regarding your behavior?* • *Regarding your modes?*
Is there anything important you would like to say to therapists who work with this technique in the context of Schema therapy?

The interviews lasted 36 to 148 min and were conducted face-to-face. The added questions were assessed via telephone for eleven of our participants. The telephone interviews lasted between 2:34 and 24:04 min. For the five participants who could not be reached we used only the existing data.

The interviews were audio-recorded and transcribed using the protocol of Dresing and Pehl (13), a system of rules for the systematic transcription of audio or video data.

### 2.3. Data analysis

Interview data were analyzed following the procedures of qualitative content analysis (QCA) (14, 15) using MAXQDA software (16). The goal of QCA is to obtain meaningful data that systematically describes the material (e.g., interviews) in order to answer a specific research question. It can be used especially when the meaning of the data is not obvious. We chose QCA because it is oriented to the research questions, and it is able to process the amounts of data produced by qualitative interviews and reduces the information the interviews contain to their core. We took a mixed approach consisting of inductive elements (not forming a priori categories) and deductive elements (framework set by the interview questions) to create a system of categories and subcategories. We started the process of data analysis by choosing six interviews with participants with different age and treatment duration, and which were conducted by different interviewers. These interviews were independently analyzed by the first author and a Master's student in psychology. Reading the interviews helped to gain familiarity with the material, no coding categories were developed a priori. In the next steps all passages which were considered relevant for answering the research questions were marked in MAXQDA using different colors. The six interviews were processed separately for the different research questions. For the transparency and traceability of the process, a coding framework was developed. In the next step of data reduction, the marked text passages were paraphrased and then generalized with a low abstraction level. Meaningless paraphrases were deleted, paraphrases with the same meaning were combined, and paraphrases with similar meanings were expressed by a new statement. In the next step a preliminary category system was established, attributing codes to the relevant text passages. Units of analysis were defined as follows: Code unit: any contextual utterance of the participant, including non-verbal utterances; context unit: a passage until the change of speaker; unit of analysis: all available interviews. At the same time, it was verified that the actual statements of the participants were captured, and the text passages were of relevance to the research question. The developed categories were sorted further, summarized, or divided if necessary and arranged hierarchically.

The category system was then presented and discussed in an expert group (including DB, AS, NA and EF) with the aim to contribute further expertise to the formation of the categories, checking them for plausibility and agreeing on the categories by discourse. After adapting suggestions of the expert group, two authors (AJ, AS) each coded five transcripts, chosen to represent participants with differences in gender, age and symptom severity. Subsequently the two datasets were exported and merged on MAXQDA to calculate the inter-rater agreement, or Cohen's Kappa (17), and found it to be κ = 0.84 which indicates a good inter-rater reliability (17).

In the next step the remaining 24 interviews were analyzed. Lastly, in consultation with the expert group, one of the authors (AJ) compiled a final adaption of the category system in which categories were summarized and new categories were integrated if adequate.

## 3. Results

A content analysis of patient's reported experiences resulted in five key domains, showing a chronological process, beginning with the initial engagement in chairwork (Domain A), over the application, including helpful and hindering aspects (Domain B), to the debriefing of the chair dialogue in the same session (Domain C) and short-term effects of chairwork (Domain D) as well as long term effects of chairwork (Domain E). This paper contains the phenomenological description of the data based on the statements of the interviewed participants. For the sake of clarity and readability we decided not the report all sub(sub)themes, but a complete overview of the category system can be found in Table 3.

**Table 3:** Category system.

**Domains/themes/subthemes/subthemes**	***N*** **(%)**
**A. Initial engagement in chairwork**	**23 (79%)**
**A 1. Initial engagement in chairwork is difficult**	**19 (66%)**
A 1.1. Problems to take the technique seriously at first	4 (14%)
A 1.2. Fear that emotions would come up hindering engagement in chairwork at first	5 (17%)
A 1.3. Difficulties taking on the perspective of specific modes	7 (24%)
A 1.4 It takes time to understand and trust the technique	11 (38%)
A 1.5 Unable to engage in chairwork for reasons that couldn't be specified	3 (10%)
**A 2. The engagement in chairwork was facilitated by a short explanation of the technique**	**13 (45%)**
A 2.1. An explanation ahead of the technique is helpful	8 (28%)
A 2.2. A short preparation phase was helpful	6 (21%)
**B. Application of chairwork**	**29 (100%)**
**B1 Factors that hindered chairwork**	**20 (69%)**
B1.1. Hindering therapist behaviors	10 (35%)
B1.1.1. Too much pressure to engage in chairwork B1.1.2. Therapist does not provide enough emotional support during chairwork	3 (10%) 3 (10%)
B1.1.3. Technique is used too often or too seldom to benefit from it	5 (17%)
B1.2. Hindering external factors	5 (17%)
B1.2.1. Noise or Interruptions	3 (10%)
B1.2.2. Restricted facilities	2 (7%)
B1.3. Hindering internal factors	13 (45%)
B1.3.1. Feeling embarrassed or ashamed	11 (38%)
B1.3.2. Punitive Parent mode hindered chairwork	3 (10%)
B1.3.3. Hard to accept how therapist treats Vulnerable Child mode	3 (10%)
B1.3.4. No need to switch chairs	3 (10%)
**B2. Specific therapist behaviors facilitate chairwork**	**27 (93%)**
B2.1. Therapist clearly guides through the process	15 (52%)
B2.1.1. Therapist restricts flood of words	3 (10%)
B2.1.2. Therapist structures process	8 (28%)
B2.1.3. Therapist stays persistent	4 (14%)
B2.1.4. Therapist provides assistance in visualizing the Vulnerable Child mode	5 (17%)
B2.2. Therapist is flexible in applying the technique	8 (28%)
B2.2.1. Therapist is flexible in applying the technique according to needs and wishes	5 (17%)
B2.2.2. Therapist gives enough time	2 (7%)
B2.2.3. Therapist stresses voluntariness	3 (10%)
B2.3. Therapist provides safety, warmth, and encouragement	26 (90%)
B2.3.1. Therapist is empathic and dedicated	19 (66%)
B2.3.2. Therapist suggests helpful mode messages	14 (48%)
B2.3.3. Therapist provides feelings of safety and support	11 (38%)
B2.3.4. Therapist restricts Punitive Parent mode	4 (14%)
B2.3.5. Therapist treats modes seriously	3 (10%)
B2.3.6. Therapist is physically close during chairwork	3 (10%)
**C. Post-processing is important**	**24 (83%)**
C 1. Therapist takes enough time for post-processing	18 (62%)
C 2. Summing up results of chairwork is essential	7 (24%)
C 3. Planning an activity after session helps to reduce distress	3 (10%)
D. Patients experienced various short-term effects	**20 (69%)**
D 1. Emotional pain right after chairwork	15 (52%)
D 2. Relieved right after chairwork	9 (31%)
D 3. Better self understanding	4 (14%)
E. Patients experienced many positive long-term effects	**29 (100%)**
E 1. Emotional experiences changed into a positive direction	**15 (52%)**
E 1.1. Improved awareness of emotions	8 (28%)
E 1.2. Emotions perceived as less overwhelming	11 (38%)
E 1.3. Less guilt and shame	2 (7%)
**E 2. Dealing differently with needs**	**7 (24%)**
E2.1 More awareness of needs	6 (21%)
E2.2 Better fulfillment of needs	4 (14%)
**E 3. Improved social skills**	**23 (79%)**
E 3.1 More openness in relationships, and improved expression of feelings and needs	16 (55%)
E 3.2. It's easier to take other's perspective	10 (35%)
E 3.3. Improved setting boundaries	4 (14%)
E 3.4. Less aggressive in relationships	11 (38%)
**E 4. Thought processes changed**	**19 (66%)**
E 4.1. Less chaos in head, more clarity regarding thoughts E 4.2. Reduced influence of dysfunctional thoughts E 4.3. Able to think before acting	16 (55%) • 11 (38%) • 4 (14%)
**E 5. Warmer toward oneself**	**9 (31%)**
E 5.1. More self-compassion	7 (24%)
E 5.2. Better self-acceptance	4 (14%)
**E 6. Changes in experiencing the mode model**	**27 (93%)**
E 6.1. More awareness of modes	17 (59%)
E 6.2. Chairwork helps to better understand and experience the schema mode model	7 (24%)
E 6.3. Improved perspective taking of the modes	11 (38%)
E 6.4. More often in Healthy Adult mode	7 (24%)
E 6.5. Reduced coping modes or coping behavior	11 (38%)
E 6.6. Less often in the Punitive Parent mode	8 (28%)
E 6.7. Different behavior toward the Vulnerable Child mode	15 (52%)
**E 7. Other positive effects**	**15 (52%)**
E 7.1. Better physical wellbeing	10 (35%)
E 7.2. More relaxed in difficult situations	10 (35%)
E 7.3. More positive in general	3 (10%)

### 3.1. Domain A Initial engagement in chairwork

Twenty-three participants (79%) reported about their initial engagement in chairwork. They described both difficulties and helpful factors, as the themes in this section show:

**Theme A.1: Initial engagement in chairwork is difficult**
Subtheme A1.1 problems to take the technique seriously at first.
Subtheme A1.1 Problems to take the technique seriously at first: Four participants (14%) reported initial difficulties in taking the chairwork seriously. They found that it “*sounds quite strange”* (P11). “*At first, I thought this is possibly nonsense and it was difficult to make sense of it”* (P15).
Subtheme A1.2 Fear that emotions would come up hindering engagement in chairwork at first: Five participants (17%) stated that they were afraid of emotions which made them hesitate to get into chairwork:

“*But at the very beginning there was also [...] fear, [...]. And I was so afraid and I wanted to protect myself from getting involved as we did a chair dialogue”* (P8).

Subtheme A1.3 Difficulties taking on the perspective of specific modes: Seven participants (24%) described that difficulties with developing an awareness for their modes and taking over the modes' perspective was a barrier in the beginning. Difficulties included “*getting into it [the mode]”* (P12), “*to stay in the mode and not switch [between modes]”* (P18) and to consciously “*switch [between the modes] if you are told to do it [by the therapist]”* (P4).

Subtheme A1.4 It takes time to understand and trust the technique: Furthermore, eleven participants (38%) described that they had trouble believing in the technique and were impatient at first:

“*The initial period was very difficult. I always thought: ‘Why am I doing this?' and ‘Why do I need this? Nothing changes anyway'. [...] So, [I was] impatient, well, it just takes time to detect any changes at all. It just doesn't happen overnight”* (P1).

The participants described how the therapist helped them to overcome the initial barrier:

“*Well, in the introduction I didn't understand at all what he wanted from me. That was one of the formative events. I stood in front of it and thought to myself: ‘What is this now?' So, it took him a little bit of time and patience to kind of explain and illustrate the whole thing to me, and that was kind of the biggest hurdle in the beginning”* (P23).

**Theme A.2: The engagement in chairwork was facilitated by a short explanation of the technique**

Thirteen participants (45%) described that they benefited from a brief introductory explanation and then getting started with chairwork quickly:

“*It was helpful that she [the therapist] explained very shortly what I had to expect [in chairwork] and then said: ‘Don't let us talk about it at length, let's try it”'* (P23).

### 3.2. Domain B Application of chairwork

All participants described helpful and hindering factors during the application of chairwork:

**Theme B.1: Factors that hindered chairwork**

Subtheme B1.1 Hindering therapist behaviors:

*Subsubtheme B.1.1.1 Too much pressure to engage in chairwork:* Three participants (10%) described that they felt pressured to continue with chairwork against their will:

“*I sometimes had the feeling that when I had reached my breaking point and said, 'No, no more,' my therapist would say, 'We still have 5 min, we can go on.”'* (P23).

“*Less helpful was [that] I had the feeling, that my therapist wanted to go through with it by hook or by crook”* (P28).

*Subsubtheme B.1.1.2 Therapist does not provide enough emotional support during chairwork: Three* participants (10%) felt “*too little emotional support”* (P13) during chairwork and wished their therapist would have been “*a bit more empathetic”* (P1).

Subtheme B1.2 Hindering external factors (e.g., noise, interruptions or restricted facilities):

Five participants (17%) reported that they felt disrupted by noise or other interruptions, like phone calls, construction noise or people stepping into the therapy room and restricted facilities:

“*Noise is always a factor. [...] If you can't concentrate properly, you're out of it very quickly”* (P3).

“*At some point there were simply not enough chairs, or it was all the same chairs”* (P9).

Subtheme B1.3 Hindering internal factors:

*Subsubtheme B.1.3.1 Feeling embarrassed or ashamed:* Eleven participants (38%) reported that a feeling of embarrassment or shame hindered them during chairwork:

“*At the outset I always thought it was a little bit embarrassing”* (P8).

Seven participants described “*strong feelings of shame”* (P19) and seven participants described experiencing devaluating thoughts about chairwork e.g.,:

“*It [chairwork] is very silly”* (P20).

“*I don't want to talk to chairs. I don't want my therapist to talk to chairs. I simply just can't take it seriously”* (P13).

Interestingly, two participants described both.

*Subsubtheme B.1.3.2 Punitive Parent Mode hindered chairwork:* Three participants (10%) explicitly described that the activation of the Punitive Parent Mode hindered them during chairwork: “*Mainly the Punitive Parent Mode kept interfering again and again. And then I couldn't quite put myself in the others' [modes] shoes”* (P11).

*Subsubtheme B.1.3.3 Hard to accept how therapist treats Vulnerable Child Mode:* Three participants (10%) described that the soft way the therapist treated the Vulnerable Child Mode was difficult to accept for them:

“*Now don't be sad, little [participants given name], or something. And that is of course something that can be both good if you get involved in it, but which can also seem a bit silly [...] I still can't quite accept it, I have to say”* (P29).

**Theme B.2: Specific therapist behaviors facilitate chairwork**

Subtheme B2.1 Therapist clearly guides through the process:

*Subsubtheme B.2.1.1 Therapist restricts flood of words:* Three participants (10%) said that it was helpful for them that the therapists restricted their speech on some occasions:

“*Actually, they always did that quite well… with my torrent of words. They have limited it well”* (P25).

“*Actually, they always did that quite well… with my torrent of words. They have limited it well”* (P25).

And further stated that they got better at accepting being stopped:

“*Since [I've] learned why they [the therapists] say that. I always remember that when the feelings of hurt come and it's helpful to accept that”* (P25).

*Subsubtheme B.2.1.2 Therapist structures process:* Eight participants (28%) described it as helpful that the therapist clearly structured the process of chairwork:

“*Well, that they [the therapists] are practically like that. Yes always directing [the process was helpful]”* (P3).

“*And for me it was good [...] that she asked me many questions and then somehow helped me get to the point”* (P19).

“*We always had limited time in the sessions, and it was always the case that my therapist was very good, made sure that the whole thing [the chairwork] was given a reasonable time frame” (*P23).

*Subsubtheme B.2.1.3 Therapist stays persistent:* Four participants (14%) stated that it helped them to engage in chairwork when their therapist insisted to use chairwork:

“*I think his insistence was helpful”* (P10).

*Subsubtheme B.2.1.4 Therapist provides assistance in visualizing the Vulnerable Child Mode:* Five participants (17%) stated that their therapist helped them to visualize their Vulnerable Child Mode by using plushies or childhood pictures for example:

“*My therapist always had a little lion sitting on a chair. That is definitely helpful in any case, so that you know, aha, there's little [participant's given name]”* (P23).

Subtheme B2.2 Therapist is flexible in applying the technique according to needs and wishes:

Eight participants (28%) reported that the therapist's flexible handling of chairwork was helpful to them, for example using current situations for chairwork instead of following a prefabricated plan:

“*Yes, it was often the case that my therapist didn't say we're going to do this somehow stubbornly but did it according to the situation. When I came with a current situation, he would bring in a chair”* (P26), or scheduling enough time for the conduct of chairwork:

“*[The therapist] gave me time, so I didn't feel stressed out, but had always enough time, to get into the roles emotionally”* (P15).

Subtheme B2.3 Therapist provides safety, warmth, and encouragement:

*Subsubtheme B.2.3.1 Therapist is empathetic and dedicated:* Nineteen participants (66%) highlighted the empathetic and committed attitude of their therapists to be helpful for them:

“*[The therapist] had a great understanding and was then able to help me to engage with it (chairwork) better”* (P15).

“*They were benevolent toward us. Sympathetic. Yes, very empathetic”* (P2).

Some expressed relief that their therapists did not expect them to “*do it [chairwork] perfectly right away”* (P29).

*Subsubtheme B.2.3.2. Therapist suggests helpful mode messages:* Fourteen participants (48%) said that it helped them with chairwork when their therapists' suggested messages for the different modes when they were stuck. Participants stated that the therapists “*helped out”* when they “*didn't know what to say in the Healthy Adult chair“* (P12):

“*So, when I didn't know what to say to either the Demanding Parent Mode or the Child Mode, she sort of jumped in and made suggestions about what I could say”* (P23).

*Subsubtheme B.2.3.3 Therapist provides feelings of safety and support:* Eleven participants (38%) stated that their therapists supported them and made them feel safe during chairwork e.g., by giving encouragement and praise:

“*Well, to convey a sense of safety, so you are really able to engage [in chairwork] without worries“* (P22).

“*Maybe to get some praise during the dialogue: 'Hey, you've done that well now and you've overcome yourself”'* (P18).

*Subsubtheme B.2.3.4 Therapist restricts Punitive Parent Mode:* Four participants (14%) reported that they found it helpful that the therapists “*put the brakes”* (P23) on the Punitive Parent Mode, only giving it “*little space”* (P24) and allowing it to be there “*only very briefly”* (P24).

*Subsubtheme B.2.3.5 Therapist treats modes seriously:* Three participants (10%) described that it helped them that therapists treated the modes seriously:

“*The absolute seriousness was helpful. Therapists are really good at it eventually. Although I actually thought, ‘what is she doing there', she talked seriously with the chairs and was totally authentic”* (P13).

*Subsubtheme B.2.3.6. Therapist is physically close during chairwork:* Three participants (10%) stated that it helped them during chairwork that the therapist stayed physically close to them, kneeling, or sitting close to their chair in general, especially when working with the Vulnerable Child Mode:

“*She was always crouched at my side during the chair dialogues”* (P23).

“*And that the therapist, was always by my side. [...] so you didn't feel alone, because when you're a little child you're also afraid, and she always gave you… always stood by you”* (P29).

### 3.3. Domain C Post-processing is important

Twenty-four participants (83%) reported that post-processing was important to them, as shown in the following themes:

**Theme C.1: Therapist takes enough time for post-processing**

Eighteen (62%) participants reported that structuring the session in such a way that enough time for post-processing remains at its end, was an important and helpful aspect:

“*Well, for me it was always important, that we talked about the chairwork afterwards”* (P22).

**Theme C.2: Summing up results of chairwork is essential**

To sum up and analyze the findings and implications of the chairwork was described as important by almost one quarter of the participants (24%):

“*During the post-processing [...] she summed up the results, what just occurred [...] That was good for me”* (P23).

**Theme C.3: Planning an activity after the session helps to reduce distress**

Three participants (10%) stated that planning something pleasant after the session was a helpful factor for them:

“*My therapist has always made sure after a difficult session that we discussed together what I can do after the therapy. Well, that I don't lie under the blanket at home, but to undertake something, visiting friends, drinking coffee [...]”* (P29).

### 3.4. Domain D Patients experienced various short-term effects

Twenty participants (69%) experienced short-term effects directly after chairwork, as described in the following themes:

**Theme D.1: Emotional pain right after chairwork**

Fifteen participants (52%) stressed that chairwork “*in the short term”* (P18) left them feeling “*agitated”* (P18) and “*extremely distressed”* (P21).

“*Afterwards you are very emotional and agitated and you find it difficult after this [chair] dialogue to let it sink in a bit and to take this intensity out of it”* (P15).

**Theme D.2: Relieved right after chairwork**

On the other side, almost one third of the participants (31%) described experiencing “*a bit of relief* ” (P23) right after the process and a positive “*change of emotions”* (P12), e.g.:

“*When I was sad before, then most of the times I wasn't so sad afterwards or whichever feeling [...]”* (P12).

**Theme D.3: Better self understanding**

Four participants (14%) also stated that they had a clearer view of themselves directly after the technique:

“*For me, the result was that I was able to understand myself better”* (P8).

### 3.5. Domain E Patients experienced many positive long-term effects

All participants reported positive long-term effects. The observed effects were wide-ranging, as can be seen in the themes of this domain:

**Theme E.1: Emotional experiences changed into a positive direction**

Subtheme E1.1 Improved awareness of emotions: Eight participants (28%) reported an improved ability to feel and name their emotions:

“*Yes, it just helped me to perceive emotions”* (P22).

“*Now I know which emotions I have this minute, if it is sadness, fury, or anything else. Yes, it helped me to perceive emotions”* (P29).

Subtheme E1.2 Emotions perceived as less overwhelming: Eleven participants (38%) reported feeling less overwhelmed by their emotions in general:

“*This alone helps a little bit by the processing [of emotions], well it is not so overwhelming”* (P23).

Others reported a reduction of specific emotions:

“*I am a little more balanced. Not so angry anymore”* (P10).

Subtheme E1.3 Less guilt and shame: Two participants (7%) attributed experiencing less “*feelings of guilt”* (P25) and “*shame”* (P25) to a long-term effect of chairwork:

“*It becomes clear to me, that it is not my fault”* (P23).

**Theme E.2: Dealing differently with needs**

Subtheme E.2.1 More awareness of needs: Six participants (21%) stated that their awareness for their needs has grown due to chairwork:

“*I understood what I needed at that moment. Or simply what I was missing”* (P5).

Here, a better awareness of the modes they were in and linking these modes to the specific need of the moment played an important role for some participants:

“*I get a clearer picture of which parts feel neglected at the moment, yes, or have some need at the moment that is not fulfilled. Or [which modes] are crying out for something, and I have a better idea of what it is really about for me”* (P18).

Subtheme E.2.2 Better fulfillment of needs: Four participants (14%) said that their ability to meet their needs improved through chairwork:

“*I myself can also fulfil more of my own needs”* (P26).

**Theme E.3: Improved social skills**

Subtheme E3.1 More openness in relationships, and improved expression of feelings and needs: More than half of the participants (55%) realized that they could better open up to others, especially that they improved their ability to express their own needs, wishes and emotions:

“*Yes, I am able to express clearer what I want and what I feel”* (P12).

“*And there are also situations where I can't cope on my own, and then I can also say, oh, sweetie, look, I need you right now, or give me a hug. I can express myself and say that, and I don't need to be ashamed, because it's something quite normal”* (P26).

Subtheme E3.2 It's easier to take other's perspective:

Ten participants (35%) described that they were able to take the view of other people, e.g., parents, partners, or friends, more easily:

“*[The change is] mainly related to my parents. [...] some things that I accused them of. I got a better understanding for them through the chairwork”* (P22).

They said that they were “*virtually being able to understand others' decisions better”* (P22).

Subtheme E3.3 Improved setting of boundaries: Four participants (14%) reported to be able to set boundaries in interpersonal relationships more easily:

“*I have learned to say no on some occasions. To think of myself and say: ‘No, I'm not going to do that today. I want to do something for myself.' And that has changed a lot”* (P1).

“*That one no longer allows oneself to be exploited”* (P26).

Subtheme E3.4 Less aggressive in relationships: Moreover, almost 40% of the participants observed being less aggressive in their relationships, that they no longer “*destroy relationships”* (P29) or “*start fights”* (P29) all the time:

“*When I am in a Coping Mode that is immediately associated with a challenge, with hatred, anger and aggression and by now I am thinking about deescalating the situation, so I am less aggressive, less angry, feeling less hatred”* (P27).

**Theme E.4: Thought processes changed**

Two thirds of participants (66%) reported changes in the way they deal with their thoughts. These improvements could be linked to three subthemes:

Subtheme E4.1 Less chaos in head, more clarity of thoughts: Sixteen participants (55%) stated experiencing their thought processes were less chaotic and somehow “clearer”:

“*A little awakening, [...] a clarity [regarding my thoughts] a real clarity, actually, that you suddenly had”* (P7).

“*And I was also clearer in what I think or what I want to say”* (P12).

Subtheme E4.2 Reduced influence of dysfunctional thoughts: Eleven participants (38%) described that they were able to reevaluate their thoughts and do a “*reality check”* (P21):

“*So, I don't listen to punishing thoughts so much anymore. I can block out not-so-sensible thoughts better. And, yeah. They don't influence me as much anymore”* (P4).

Subtheme E4.3 Able to think before acting: Four participants (14%) were able to think before they acted:

“*Well, in the long term it's always that I no longer overshoot the mark like that, but that I really think things through carefully. And then also weigh the situation with pros and cons”* (P3).

**Theme E.5: Warmer toward oneself**

Subtheme E5.1 More self-compassion: Seven participants (24%) described being more self-compassionate due to chairwork:

“*[I developed a] much more loving interaction and also, an understanding for myself”* (P7).

“*I'm learning to love myself”* (P1).

Subtheme E5.2 Better self-acceptance: Four participants (14%) stated that they were able to be more accepting toward themselves:

“*I just [gained more] self-acceptance*” (P6).

“*[I realized that] I'm allowed to make mistakes*” (P25).

**Theme E.6: Changes in experiencing the mode model**

Almost all participants (93%) described experiencing changes in their mode model ranging from more awareness for their modes and taking over a mode's perspective more easily to a deeper understanding and experiencing of the mode model through chairwork. Furthermore, they described being able to spend more time in the Healthy Adult Mode and less time in the Punitive Parent and in Coping Modes, as well as a changed attitude toward the Vulnerable Child Mode:

Subtheme E6.1 More awareness of modes: **Seventeen** participants (59%) stated that chairwork helped them to sharpen their awareness for the occurrence of different modes:

“*To make [the modes] a little bit more visible and also to be more aware of the modes. I found that helpful”* (P14).

“*Exactly, that I have learned a lot through this [chairwork], to reflect myself now. In which mode am I now really, which I didn't know before”* (P5).

Subtheme E6.2 Chairwork helps to better understand and experience the schema mode model: **Seven** participants (24%) described that chairwork helped them to understand and experience their schema mode model more thoroughly:

“*[Chairwork worked] better than in the imagination. Because that's where I really, felt the mode changes in chair therapy. While I was on one chair, I noticed that I was already changing to the next one and then I moved on my own”* (P5).

“*The technique helped me not only to perceive it visually, but also to feel it, the mode model. And yes, that helped me a lot”* (P22).

Subtheme E6.3 Improved perspective taking of the modes: **Eleven** participants (38%) stated that chairwork helped them to take over the perspective of their modes more easily:

“*So, the advantage [of chairwork] was somehow this change of perspective”* (P17).

“*Yes, as I said, that one has conversations with oneself, with one's own modes, that you can also see them as persons, not as enemies”* (P25).

Subtheme E6.4 More often in Healthy Adult Mode: **Seven** participants (24%) noticed that they use their Healthy Adult Mode more frequently:

“*And then [after the chairwork I was] very much in my Adult, Healthy Adult”* (P21).

“*Yes, I also try to listen to my Healthy Adult more often”* (P23).

Subtheme E6.5 Reduced Coping Modes or coping behavior: **Eleven** participants (38%) reported experiencing fewer Coping Modes, for example they described less dissociation: “*I no longer dissociate very often”* (P24), reduced self-harm: “*There are no more [self-inflicted] injuries; this mindset is gone too”* (P11), or binge eating: “*The binge eating has become less and is also less rampant”* (P20).

“*I no longer follow the impulses that the Coping Modes suggests to me. I am simply more aware of it, by knowing what it is. I'm more aware of it, I know what it is, why it comes up and I decide against it. And therefore, behave differently than I would have done in the past”* (P 23).

Subtheme E6.6 Less often in the Punitive Parent Mode: Almost one third of the participants (28%) stated that chairwork helped them to reduce and stop their Punitive Parent Mode. Three participants explicitly indicated that the ”throwing out“ of the chair of the Punitive Parent Mode together with the therapist during chairwork particularly impressed them and that they were able to use this experience to limit Punitive Parent Mode later:

“*That was a very positive experience for me. Because since then I also try not to let this Punishing Mode into my apartment. Instead, I have a box in front of my front door, where I usually put garden pads. And in my mind [...] I leave the Demanding, Punishing Mode out there. It then goes into the box, and I say, ‘You're not coming into my apartment.' And that has worked pretty well”* (P3).

“*For example, there was a situation where we simply took the chair of the Punishing Mode and just carried it out and put it outside the door. And that helped me, to try to do that emotionally, and to try to do that by myself”* (P22).

Subtheme E6.7 Different behavior toward the Vulnerable Child Mode: More than half of the participants (52%) stated that chairwork helped them to allow their Vulnerable Child Mode to be there and that they could better soothe and take care for it:

“*So, sometimes I feel sorry for this child and say to myself: ‘I'm really sorry”'* (P8).

“*Yes, for example, I speak to little [participant's given name]. I tell her that everything is okay, that it's totally okay that she's sad right now”* (P29).

“*It has also happened that I went for a walk afterwards and then, once, I suddenly saw my little child jumping in front of me and thought, ‘Okay, you may enjoy yourself. You can have fun, you can be happy now. Come hold my hand and I'll take care of you.' That felt pretty good”* (P9).

**Theme E.7: Other positive effects**

Subtheme E7.1 Better physical wellbeing: Ten participants (35%) noticed an improvement of their physical wellbeing like more relaxation and less pain:

“*And I would also say that I am also physically more relaxed”* (P26).

“*[I experience] less back pain [and I am] less tense”* (P21).

Subtheme E7.2 More relaxed in difficult situations: Ten participants (35%) observed being more relaxed in difficult situations:

“*So now I can just be more relaxed about it and so on”* (P21).

“*It doesn't stress me when I somehow see something that is actually [...] that I wouldn't have been able to do a year or two ago. [...] simply doesn't stress me as much anymore”* (P24).

Subtheme E7.3 More positive in general: Three participants (10%) described that chairwork helped them to get “*simply more positive, in general”* (P21).

Overall, when patients were asked to weigh the costs and benefits of chairwork, the vast majority of participants (83%) felt that the benefits of chairwork outweighed the costs, with only one participant stating that the costs of chairwork were greater than the benefits.

## 4. Discussion

In this study we aimed to gain insights into the experiences of patients with BPD with chairwork as a part of their ST treatment, to explore which factors participants experienced as helpful or hindering during chairwork, and which short- and long-term effects participants experienced after receiving chairwork. A qualitative content analysis of patient's reported experiences resulted in five key domains along a timeline starting with the preparation and first experiences with chairwork, over the implementation including several hindering and facilitating factors, to the post-processing and short- as well as long-term effects. The findings highlighted that patients found engaging in chairwork difficult in the beginning, and revealed for each part of the process difficulties and hindering factors as well as several helpful factors to overcome difficulties. Regarding the effects of chairwork, approximately half of the patients observed emotional pain right after chairwork, but all patients reported positive effects in the long run including favorable changes in their emotional experiences, their thought processes, a better understanding of the mode model, positive mode changes, improved social skills and more feelings of compassion, acceptance, and forgiveness toward oneself.

### 4.1. Difficulties and specific schema modes hindering chairwork

Two thirds of participants reported difficulties in their initial engagement with chairwork. Barriers included difficulties taking the method seriously at first, fear of emotions coming up, and difficulties taking the perspective of the modes in the beginning. In a qualitative study looking into how recovery in BPD occurs, Katsakou and colleagues (18), correspondingly found that fighting ambivalence and committing to action was one of three key recovery processes.

Almost half of our participants reported internal factors which hindered their engagement in chairwork. These factors included feelings of ridiculousness persisting during the implementation of chairwork, being hindered by one's Punitive Parent Mode and difficulties to accept how the therapist treats the Vulnerable Child Mode.

In the first study of our research series Schaich and colleagues (7) interviewed a slightly smaller sample of the same patients. Correspondingly to our findings, the participants described that an unspecified internal blockage hindered their engagement in IR and that they had difficulty taking IR seriously. This might indicate that some patients fear experiential techniques, feel ashamed and/or have strong avoidance coping modes which should be targeted early in treatment in order to help patients use these treatment techniques and maximize their profit from therapy. This effect seems to be not limited to chairwork in ST.

In line with our results Stiegler and colleagues (19) reported in their qualitative study on two-chair dialogues in Emotion-Focused Therapy (EFT) that the participants scoring high on self-criticism often experienced embarrassment or awkwardness before they decided to engage in chairwork. The participants described the technique as intense and demanding, but also meaningful. However, only few participants found it too intense to be useful. Participants with depression receiving Compassion-Focused Therapy (CFT) described experiencing various intense emotions during chairwork (20).

While the majority of the interviewees were able to shift in and out of emotions by moving chairs, some participants experienced the emotions as overwhelming. Most participants in our study on chairwork as well as in our study on IR were able to overcome this hindrance later on and rated the benefits of the technique greater than the costs. Thus, therapists performing chairwork or other experiential techniques should not be discouraged if patients show resistance at the beginning. Interestingly, the exposure to formerly avoided painful emotions was connected to better outcome in participants with BPD receiving either Dialectical Behavior Therapy (DBT) or Mentalization-Based Therapy (MBT) in a qualitative study by Barnicot and colleagues (21). This might also be an important factor in ST.

More than two thirds of the participants pointed out factors hindering the successful implementation of chairwork. A few patients described external factors, including noise, interruptions and restricted facilities that hindered their engagement in chairwork. In particular, the feeling of being disturbed by noise is consistent with the findings of Schaich and colleagues (7). Restricted facilities (e.g., little space and lack of chairs) was also mentioned as hindering in the study of Pugh and colleagues (22). One third of our participants also described hindering therapists' behavior. Here, participants felt too much pressure to engage in chairwork or did not feel sufficiently supported by the therapist.

The participants appreciated when the therapists were flexible in applying chair dialogues according to their needs and wishes. This is in accordance with a qualitative study of Muntigl and colleagues (23) looking into the participants' reluctance to engage in chairwork in EFT. The participants in this study described helpful therapists' behaviors, such as offering alternatives, providing an extended rationale for chairwork and elaborating on the proposals to overcome barriers to engage in chairwork. The authors concluded that a flexible and responsive approach of “collaborative negotiations” was most likely to help participants to engage in chairwork.

### 4.2. Factors facilitating chairwork

Almost all participants reported specific therapist's behaviors they found helpful while engaging in chairwork. Facilitating factors reported included being provided with safety, warmth, and encouragement by the therapist, and being guided through the process of chairwork. De Klerk and colleagues (5) reported correspondingly that their participants, patients with other PDs receiving ST as well as their therapists conducting ST, described the therapeutic relationship in general as a helpful factor in ST. In the study of Tan and colleagues (6), participants with BPD receiving ST also reported the extent of support by their therapist and feeling emotionally connected as essential. In line with these findings, a meta-analysis of 14 qualitative studies showed that helpful characteristics of the treatment and the therapeutic relationship in the view of patients with BPD receiving psychological therapy or generic mental health services, included safety and containment, as well as being cared for and respected (24).

Schaich and colleagues (7) describe, in line with our findings, that participants value if therapists can adjust flexibly to patients' needs and provide safety and contact. For our participants it was especially important that therapists were empathetic and dedicated during chairwork, suggested helpful messages, stopped the Punitive Parent Mode and were physically close and supportive especially when working with the Vulnerable Child Mode. Therapists' use of “process skills” (25) or “deepening techniques” (26) are described as important factors to ensure chairwork is effective. Some of these skills were described by our participants as helpful factors, too, including “feeding a sentence” (27), modeling and praise/encouragement.

These statements fit well with the concept of the therapeutic relationship in ST, “limited re-parenting,” in which the therapist within the boundaries of a professional therapy relationship behaves like a “good parent” toward the patient and follows specific goals toward the respective modes (28–30). Meta-analyses have found a moderate but stable relationship between therapeutic alliance and therapeutic outcome across a wide range of different treatments (31, 32). Correspondingly, facilitating therapist behavior was the largest helpful factor participants described in our study; most of our participants described it as helpful that their therapist provided them safety, warmth, and encouragement.

As in the other qualitative studies on ST in general (5, 6) and the study on participants' experiences in IR (7), participants in our study stated that taking enough time for post-processing after experiential exercises was important.

### 4.3. Effects of chairwork on mode modification and other factors

Participants described various short- and long-term effects that occurred after using chairwork in ST, which they directly associated with chairwork. More than half of the participants reported initially experiencing emotional pain, one-third described feelings of relief and a few participants described greater self-acceptance. This is in accordance with other studies in which patients described chairwork as challenging with both intense and painful emotions during the exercise but also the experience of relief, peace, and self-compassion right after chairwork (20, 33–35).

Notably, considerably more patients (more than 80%) of the participants in the study of Schaich and colleagues (7) reported overwhelming emotions during and directly after IR. Also, 19% of the patients in Schaich and colleagues (7) weighed costs of IR to be higher than the benefits, while only one patient (3%) estimated the costs higher than the benefits in our study on chairwork. Thus, it might be that patients experience IR as more emotionally demanding compared to chairwork. This is not surprising since in this qualitative research series patients with severe BPD were treated and IR focusses directly on memories of childhood trauma whereas chairwork mostly addresses difficult situations in the present. Notably, most patients [67% in Schaich et al. (7) vs. 83% in the present evaluation] valued the therapeutic effects from these techniques and weighed the benefits higher than the costs. All patients reported broad therapeutic gains as long-term effects, including improved emotional experiences, better dealing with needs, improved social skills, dealing differently with cognitive processes, being warmer toward oneself, experiencing changes in the mode model and other positive changes. The findings are in line with the other qualitative studies exploring ST or IR for (B)PD-patients (5–7).

To our knowledge there are no other qualitative studies so far, that looked closely into the modification of modes through chairwork. Regarding long-term effects, participants in our study described extensive changes regarding the schema mode model in line with the goals of ST (28), namely spending more time in the Healthy Adult Mode, reduction of the Punitive Parent and Coping Modes and changes in behavior toward the Vulnerable Child Mode. Interestingly, participants described not only spending more time in Healthy Adult Mode, but also being able to switch to Healthy Adult Mode directly during chairwork (P21). Almost a third of participants reported that chairwork helped them reduce their Punitive Parent Mode. This strengthening of the Healthy Adult Mode and reduction of the Punitive Parent Mode resembles the strengthening of the compassionate self and the reduction of the criticizing self-reported in the study of Bell and colleagues (36).

Other reported long-term changes our participants described can be hypothesized to go hand in hand with a strengthening of the Healthy Adult Mode. Almost a quarter of participants directly stated that they spend more time in the Healthy Adult Mode. But additionally, being able to better deal with emotions, changes in cognitive processes to allow more effective problem solving, handling social interactions more healthily, being more compassionate and forgiving with oneself as well as being aware of one's needs are all qualities of the Healthy Adult.

These long-term effects correspond to another qualitative study exploring BPD patients' experiences with ST (6). In this study various therapeutic gains were reported after a 2-year treatment, including increased insight, better connection with one's emotions, improved self-confidence, increased cognitive flexibility in terms of taking alternative perspectives, being less harsh to oneself and reduced internalized punitive voice, changes similar to what our participants reported. Also, in the study of Katsakou et al. (18) on other BPD treatments, areas of perceived change included self-acceptance and self-confidence, new ways of relating to others, taking control of emotions and thoughts as well as implementing practical changes and developing hope.

### 4.4. Clinical and research implications of the findings

The findings of our study allow us to make some recommendations for optimizing chairwork in clinical practice and to formulate some suggestions for future research.

#### 4.4.1. Clinical implications

Although our findings are only preliminary and need further corroboration, therapists can consider optimizing the implementation of chairwork when working with patients with BPD, based on our participants' statements.

#### 4.4.2. Dealing with dysfunctional coping modes such as the detached protector mode early in treatment

Our findings suggest that to get into chairwork, patients need to deal with and overcome feelings, e.g., shame or anxiety. Getting started quickly with the technique and gathering personal experiences with chairwork was one strategy named by our participants which helped them overcome expectation anxiety as well as feelings of shame. Therapists should not be discouraged if patients state things like “this is silly” or “I can't take this seriously” at the beginning of chairwork, but to place the reaction in the mode model (Demanding or Punitive Parent Modes, Coping Modes) and confront the patient empathically.

#### 4.4.3. Keeping the punitive and demanding parent modes at bay

It can be assumed that persisting feelings of ridiculousness and shame relate to the activation of the Punitive Parent Mode. Therefore, helping patients to reduce their Punitive Parent Mode is particularly important. Addressing and overcoming these barriers seems particularly promising because our participants described that they profited from their therapist restricting the Punitive Parent during chairwork, as well as being able to control the Punitive Parent better themselves afterward. Also, managing expectations by making it clear that it is not necessary to master the technique perfectly from the get go was described as a helpful therapist behavior.

#### 4.4.4. Providing safety and warmth for the vulnerable child mode

Therapists can support patients by providing a sense of warmth and safety through limited reparenting. Our participants described that suggesting helpful messages, stopping the Punitive Parent, and physical closeness of the therapist during chairwork, e.g., sitting or squatting next to them, provided a feeling of safety and being comforted when they were in the Vulnerable Child Mode. The use of plushies or photographs to visualize the Vulnerable Child on the chair was considered a helpful technique. Additionally, the therapists can provide favorable frame conditions by minimizing noise disturbances like phone calls and preparing enough space and chairs to comfortably conduct chairwork.

#### 4.4.5. Encouraging the healthy adult mode

A balance between persistent encouragement to engage in chairwork on the one hand and emphasizing the patient's voluntariness on the other hand was described to be helpful. Furthermore, the flexible use of chairwork to deal with current situations and problems was positively regarded. Also modeling of modes and healthy attitudes or suggesting helpful messages was valued.

#### 4.4.6. Dealing with painful emotions

Therapists should consider that patients reported painful emotions during and right after chairwork. In addition to the techniques that we mentioned above, therapist should take time to validate this emotional distress and express compassion for the associated pain. The emotional arousal should be kept tolerable (“window of tolerance”) (37), so that the patient will not be overwhelmed by emotions and new experiences can be integrated. Especially in the beginning, chairwork should be kept short (max. 20 min), so that therapist and patient have enough time to debrief and to regulate emotions.

### 4.5. Suggestion for further research

For future research it might be interesting to include more patients struggling with the technique or drop-outs to gather further data on barriers to engage successfully in chairwork and shed more light onto preventing these struggles. Two of our participants dropped out of the treatment after the interview. One of them rated the costs of chairwork higher than the benefits, the other stated the opposite. It would be interesting to interview more dropouts on their experiences because they may have interesting insight in barriers hindering the successful implementation of chairwork in particular, and the completion of therapy in general.

## 5. Limitations and strengths

As far as we know this is the first qualitative study exploring BPD patients' experiences with chairwork in detail. However, we recruited our participants by means of convenience sampling, therefore it can be hypothesized that patients discontent with ST may have been less willing to participate in this study. However, two participants described being unable to engage in chairwork and several patients described initial problems with chairwork, so we were able to gather some information about patients struggling with chairwork and the barriers they were experiencing. Because the interviews were conducted during or shortly after the treatment phase, we do not obtain information about patients' long-term experiences. Furthermore, the sample was predominantly female, and all participants received chairwork within the framework of ST in the same outpatient clinic. Due to this setting, the participants were complex BPD patients with high BPD symptom severity and comorbidity. Based on these factors, generalizability is limited. Furthermore, although the interviews started with an open question regarding the experiences of the participants with chairwork, further prompting may have led to a certain bias.

Because the participants received chairwork in the context of ST it is difficult to attribute specific effects to specific techniques delivered in a complex treatment program. Although answers indicated that participants were aware of this problem and tried to link treatment effects to chairwork as best as they could, of course therapeutic gains might be due to other ST-specific or unspecific treatment components. On the other hand, it might also be possible that some effects of chairwork were overseen and attributed to other techniques or aspects outside of the therapy room. Lastly, because all quotations were translated into English for this publication, there may be some loss of meaning.

Strengths of this study were the conduct of the interviews by interviewers not involved in the treatment process and not specially trained in ST or the conduct of chairwork either, the consensual development of the category system by two raters, the use of an intercoder agreement as well as the discussion of the category system within an expert group. We used a large sample to ensure data saturation as well as a sample varying in severity of symptoms, comorbidity, gender, age, and treatment phase. To consider varying experiences due to the stage of the therapeutic process we conducted interviews at different stages of therapy, beginning with 6 months into ST until the end of the program after 18 months.

## 6. Conclusion

In our study chairwork was experienced as a valuable experiential technique by patients with BPD receiving ST, which the participants associated with many positive meaningful short- and long-term changes. It was also described as emotionally activating and associated with the experience of emotional pain. The first engagement in chairwork took time and was difficult, but most participants were able to overcome these barriers. A short and simple preparation phase, offering enough time for the implementation of chairwork as well as debriefing, was described as helpful. Participants were able to point out various helpful and hindering factors which can be used by therapists to successfully implement chairwork. Most participants valued chairwork as a powerful technique that helped facilitate changes in various aspects of life as well as ST-specific changes regarding the modes and the schema mode model.

## Data availability statement

The datasets presented in this article are not readily available to protect the patients' privacy and ensure the secure storage of their data. Requests to access the datasets should be directed to eva.fassbinder@uksh.de or anja.schaich@uksh.de.

## Ethics statement

The studies involving human participants were reviewed and approved by Ethics Committee of Lübeck University (reference number 13-005). The patients/participants provided their written informed consent to participate in this study.

## Author contributions

AJ: drafting main body of the manuscript, revising manuscript following feedback, transcription, coding, involved in organization of logistics, data management, and recruitment of patients. AS: drafting main body of the manuscript, revising manuscript following feedback, involved in organization of logistics, data management, and recruitment of patients. DB: qualitative research counseling, involved in conception and design of the qualitative study, training of interviewers and coders in qualitative data assessment, and provided critical revision of the manuscript. NA: involved in organization of logistics, data management, recruitment of patients, and provided critical revision of the manuscript. KJ-C: involved in conception and design and implementation of the study provided critical revision of the manuscript. AA: external ST and chairwork expert, involved in the development of study protocol, development of the qualitative interview, and provided critical revision of the manuscript. US: involved in conception and design of the study, development of the study protocol, development of the qualitative interview, and provided critical revision of the manuscript. EF: coordinating investigator, initial conception and design of the study, development of the study protocol, development of the qualitative interview, substantial input to writing main body of the manuscript, and funding acquisition. Sadly, US has passed away during the final phase of the submission process for this manuscript, but other than the final approval of the manuscript, and he fulfills all criteria for co-authorship. All authors but US revised the manuscript critically and approved the final version.
